# Spray-Dried Inhalable Microparticles Combining Remdesivir and Ebselen against SARS-CoV-2 Infection

**DOI:** 10.3390/pharmaceutics15092229

**Published:** 2023-08-29

**Authors:** Tushar Saha, Shubhra Sinha, Rhodri Harfoot, Miguel E. Quiñones-Mateu, Shyamal C. Das

**Affiliations:** 1 School of Pharmacy, University of Otago, Dunedin 9054, New Zealand; tushar.saha@postgrad.otago.ac.nz; 2 Department of Microbiology and Immunology, School of Biomedical Sciences, University of Otago, Dunedin 9054, New Zealand; shubhra.sinha@otago.ac.nz (S.S.); rhodri.harfoot@otago.ac.nz (R.H.); mquinon4@uwo.ca (M.E.Q.-M.)

**Keywords:** COVID-19, SARS-CoV-2, remdesivir, ebselen, synergistic, dry powder

## Abstract

There is a continuous effort to develop efficient treatments for coronavirus disease 2019 (COVID-19) and other viral respiratory diseases. Among the different strategies, inhaled treatment is considered one of the most logical and efficient approaches to treating COVID-19, as the causative “SARS-CoV-2 virus RNA” predominantly infects the respiratory tract. COVID-19 treatments initially relied on repurposed drugs, with a few additional strategies developed during the last two years, and all of them are based on monotherapy. However, drug combinations have been found to be more effective than monotherapy in other viral diseases such as HIV, influenza, and hepatitis C virus. In the case of SARS-CoV-2 infection, in vitro studies have shown synergistic antiviral activity combining remdesivir with ebselen, an organoselenium compound. Therefore, these drug combinations could ensure better therapeutic outcomes than the individual agents. In this study, we developed a dry powder formulation containing remdesivir and ebselen using a spray-drying technique and used L-leucine as an aerosolization enhancer. The prepared dry powders were spherical and crystalline, with a mean particle size between 1 and 3 µm, indicating their suitability for inhalation. The emitted dose (ED) and fine particle fraction (FPF) of remdesivir- and ebselen-containing dry powders were ~80% and ~57% when prepared without L-leucine. The ED as well as the FPF significantly increased with values of >86% and >67%, respectively, when L-leucine was incorporated. More importantly, the single and combinational dry powder of remdesivir and ebselen showed minimal cytotoxicity (CC_50_ > 100 μM) in Calu-3 cells, retaining their anti-SARS-CoV-2 properties (EC_50_ 2.77 to 18.64 μM). In summary, we developed an inhalable dry powder combination of remdesivir and ebselen using a spray-drying technique. The spray-dried inhalable microparticles retained their limited cytotoxicity and specific antiviral properties. Future in vivo studies are needed to verify the potential use of these remdesivir/ebselen combinational spray-dried inhalable microparticles to block the SARS-CoV-2 replication in the respiratory tract.

## 1. Introduction

The viral respiratory pathogen severe acute respiratory syndrome coronavirus 2 (SARS-CoV-2) is responsible for the COVID-19 pandemic. The virus enters the host through inhalation, and causes extensive lung tissue damage, perhaps spreading to multiple organs [1,2,3,4,5,6]. This is why the respiratory tract/lung is considered as the principal target site of infection for SARS-CoV-2 [7]. There is an urgent need for efficient treatment to lessen the morbidity and mortality in the early progression of this disease [8,9,10]. The available treatments against this virus include repurposed and novel drugs, i.e., remdesivir, molnupiravir, paxlovid, monoclonal antibodies, convalescent plasma, and others [11,12,13,14].

Among the tested drugs, remdesivir, an antiviral agent, received the first U.S. FDA approval for SARS-CoV-2 infection, which was initially developed for the Ebola virus [15,16]. Remdesivir could have multiple drug–drug interactions that require specific evaluation prior to use. Also, there are reported side effects of remdesivir including liver injury, breathing problems, a low blood oxygen level, etc. [15]. Remdesivir is currently administered as an intravenous (IV) injection because the oral and intramuscular (IM) routes for delivering remdesivir are unsuitable [17]. The drug was cleared by first-pass metabolism when administered orally and showed variable release from the muscle during IM administration [17]. The current IV injection of remdesivir contains a high amount of sulfobutylether-beta-cyclodextrin (SBECD) as a solubility enhancer [17]. Because SBECD is removed by the kidney, it is not recommended for patients with renal impairment [17]. The efficacy of intravenous remdesivir has varied among reports, which limits its widespread use for the treatment of COVID-19 [18]. Furthermore, the IV administration form necessitates specific care and is typically reserved for critically ill and hospitalized patients, limiting the accessibility of this medication. This is why an easy-to-deliver and more accessible dosage form is required during the pandemic.

Since the principal route of SARS-CoV-2 infection is the respiratory tract, the delivery of antiviral agents directly to the infection site will offer several potential advantages over oral, IM, and IV routes of administration. An effective drug concentration can be obtained in the target site at a lower dose, bypassing the first-pass metabolism and minimizing the unwanted side effects [19,20,21]. The inhaled monotherapy of remdesivir against SARS-CoV-2 has been published including a dry powder inhaler and a liposomal solution for a nebulizer [17,22,23]. The inhaled remdesivir showed lower dose requirements than IV dosing and a better antiviral activity in the respiratory tract tissues of African green monkeys than the placebo [24]. These published data show the potential benefits of inhaled remdesivir over IV remdesivir.

In treating viral diseases, drug combination was found to be more effective than monotherapy, as a rightly chosen drug combination can exert a synergistic effect, and it is hard to develop resistance against combinational drugs than a single agent [25,26]. For example, the “adamantanes” group of drugs as a monotherapy has been found to be resistant to the influenza A virus, and drug combinations showed better therapeutic effects against HIV (combination antiretroviral therapy), HCV (direct antivirals), as well as influenza A-infected patients [25,26]. In the specific case of remdesivir, it has been shown to inhibit SARS-CoV-2 replication synergistically on Vero E6 cells in combination with ebselen [27]. Ebselen is a clinically safe organoselenium compound that was initially developed to treat noise-induced hearing problems. This drug has shown potent antimicrobial activity against numerous pathogens including SARS-CoV-2 [28,29,30]. The various modes of action of these drugs may account for their synergistic antiviral efficacy against SARS-CoV-2. Remdesivir inhibits the replication process, whereas ebselen acts as the main protease inhibitor [27]. As a result, combining remdesivir and ebselen will result in greater antiviral activity than either agent alone.

The above discussions provide insight into the advantages of the inhaled delivery of remdesivir and ebselen as a combination treatment for COVID-19. However, the selection of an appropriate delivery device is also a prerequisite while developing a formulation based on the infection type and disease conditions [7]. Nebulizers, dry powder inhalers (DPIs), and pressurized metered-dose inhalers (pMDls) are some of the widely used inhaled delivery devices among which nebulizers and DPIs can deliver high doses, whereas pMDIs are designed for low-dose delivery in the lung [7]. For treating infections, a high dose is generally required at the infection site [7]. DPIs and nebulizers are both suitable for high-dose delivery in the respiratory tract, although DPIs are more advantageous than nebulizers as DPIs are stable, portable, easy to administer, non-spreadable, and suitable for delivering multiple drugs [31,32]. Nebulizers, on the other hand, are mostly used for hospitalized patients and have a low delivery efficiency. As SARS-CoV-2 is a contagious virus, a special set-up is required during the nebulization of drugs to minimize its spreading [33]. All these factors make DPIs an ideal dosage form for SARS-CoV-2-infected patients, which can reduce the virus spreading as well. Spray-drying is a popular technique for producing such dry powder [34,35] because it is faster, more reproducible, and less expensive than other powder production processes [34,35].

In this study, we developed and characterized the inhalable dry powder containing remdesivir and ebselen utilizing the spray-drying technique. The in vitro safety and efficacy of the prepared dry powders were also assessed.

## 2. Materials and Methods

### 2.1. Materials

Remdesivir (purity 99%, molecular weight 602.6 g/mol), ebselen (purity 98%, molecular weight 274.18 g/mol), and L-leucine (molecular weight 131.17 g/mol) were purchased from Pure Chemistry Scientific Inc., Sugarland, TX, USA; Angel Pharmatech Ltd., Shanghai, China; and Hangzhou Dayangchem Co., Ltd., Hangzhou, China, respectively. High-Performance Liquid Chromatography (HPLC) grade acetonitrile and methanol (Merck, Darmstadt, Germany) were used in this study. The in-house purified water was used (Millipore Corporation, Burlington, MA, USA).

### 2.2. HPLC Analysis for Drug Quantification

The amounts of remdesivir and ebselen were quantified with a validated HPLC method (Agilent, Santa Clara, CA, USA) using a C18 fusion column (Synergi Fusion Column, 250 mm × 4.6 mm, 5 μm, Phenomenex, Torrance, CA, USA). The mobile phase contained acetonitrile (45%), methanol (45%), and water (10%). The injection volume was 5 μL for each sample, and the flow rate was 0.8 mL/min with an oven temperature of 30 °C. The retention time was ~4 min for remdesivir and ~5 min for ebselen, with a total run time of 10 min. A linear calibration curve (R2 > 0.999) was obtained over the 1–100 μg/mL concentration range.

### 2.3. Inhalable Dry Powder Preparation

The dry powder formulation containing remdesivir and ebselen was prepared using a Buchi B-290 spray dryer with a fixed aspiration (80%), nozzle diameter (0.7 mm), pump feeding rate (1 mL/min), drying gas flow rate (670 L/h), inlet temperature (80 °C), and feed concentration (0.8% *w*/*v*) in methanol. L-leucine, a generally recommended as safe (GRAS) amino acid, was used to reduce the powder cohesiveness for better aerosolization properties of the dry powder. The drug(s) and excipient were dissolved in methanol as per the ratio in Table 1, and the solution was spray-dried, maintaining the described parameters.

### 2.4. Yield Determination

The ratio of the obtained dry powder and the total powder used to prepare the feed solution was used to measure the powder yield (Equation (1)).

(1) $$\mathrm{Yield}=\frac{\mathrm{Obtained}\mathrm{powder}\mathrm{weight}(\mathrm{mg})}{\mathrm{Total}\mathrm{powder}\mathrm{used}\mathrm{in}\mathrm{the}\mathrm{formulation}(\mathrm{mg})}\times 100\%$$

### 2.5. Determination of Residual Solvent

The amount of residual solvent present in the dry powder formulation was investigated using a TGA Q50 analyzer (TA Instruments, New Castle, DE, USA). A specific amount (2–3 mg) of powder was loaded on the TGA pan and heated until 120 °C from room temperature (22 ± 3 °C) with a fixed temperature rise (10 °C/min). The residual solvent present in the dry powder was determined from the weight loss of the powder. An inbuilt software (Version 4.3, TRIOS) of the TGA analyzer was used to determine the weight loss. The tests were performed twice.

### 2.6. Powder Morphology and Particle Size

Scanning electron microscopy (Carl Zeiss Inc., Oberkochen, Germany) was utilized to observe and capture the image of prepared dry powders. Approximately 5 mg of powder samples (raw materials and dry powder formulations) were kept on a carbon adhesive tape using a carbon coater (Quorum Technologies Ltd., Sussex, UK) and coated with gold–palladium alloy. The images were captured, and the particle size was analyzed from the captured image by measuring the geometric diameter of no less than 300 particles. To determine the geometric diameter, ImageJ software (Version 1.53e, National Institutes of Health, Bethesda, MD, USA) was used.

### 2.7. Drug–Drug/Drug–Excipient Interaction

The interaction between the selected drugs and the excipient was analyzed using an ATR-FTIR instrument (Varian Inc., Palo Alto, CA, USA). Around 2–5 mg of samples was kept on the crystal plate, and the spectra were recorded over 500–4000 cm^–1^. The spectral resolution was set to 4 cm^–1^.

### 2.8. Powder Crystallinity

The nature of selected raw materials and dry powders were assessed through powder X-ray diffraction collected on Agilent Technologies Supernova system using Cu Kα (λ = 1.54184 Å) radiation. The powder was finely milled before being mounted on a nylon loop in a small amount of paratone-N oil. The data were recorded at 2θ from 5° to 35° with a step size of 0.010°. CrysAlis Pro software (version 1.171.35.15) was used to analyze all the data.

### 2.9. In Vitro Aerosolization Behavior

The in vitro aerosolization behavior of the dry powders was investigated using a Next-Generation Impactor (NGI, Copley Scientific Ltd., Nottingham, UK), and the tests were performed in triplicate. Around 20 mg of powder formulation was filled into the capsules (HPMC, size 3) and activated via Aerolizer (Foradil aerolizer, Novartis Pharmaceutical Ltd., London, UK). The inspiratory airflow was kept at 100 L/min to disperse the dry powder from the device over 2.4 s. The amount of powder deposited in the mouthpiece, NGI stages (S1–S7), micro-orifice collector (MOC), aerolizer, and capsule was obtained by dissolving them in acetonitrile, and quantified via liquid chromatography. The cut-off diameters of different stages (S1–S7) and the MOC of the impactor at the selected flowrate (100 L/min) are 6.12, 3.42, 2.18, 1.31, 0.72, 0.40, 0.24, and 0.07 µm, respectively [36].

The recovered dose (RD) is the entire drug quantified from the aerolizer and all the stages of NGI. Emitted dose (ED) is the amount of the drugs that is released from the device and obtained from the mouthpiece and all the stages of NGI. The fine particle dose (FPD) was calculated by interpolating the impactor data graph (cumulative mass vs. D_50_), and this is the amount of drugs in the particles with an aerodynamic diameter of ≤5 µm. The %fine particle fraction (%FPF) is the fine particle dose (FPD) expressed as corresponding to the emitted dose (ED).

### 2.10. Cellular Toxicity Assay

The cytotoxicities of remdesivir, ebselen, and spray-dried powder were assessed by quantifying both cell viability and cellular proliferation in various cell lines using the XTT [2,3-Bis-(2-Methoxy-4-Nitro-5-Sulfophenyl)-2H-Tetrazolium-5-Carboxanilide] colorimetric method from the Cell Proliferation Kit II (Merck Sigma-Aldrich, Darmstadt, Germany) as published earlier [21]. In brief, 2 × 10^4^ Calu-3 cells per well were seeded in a 96-well plate and incubated at 37 °C with 5% CO_2_ overnight. The cell culture medium (Minimum Essential Medium Eagle and 10% Fetal Bovine Serum) was replaced with fresh media containing the respective compounds at eight different concentrations. These concentrations started at 100 µM and were prepared through sequential 1:2 dilutions with the cell culture medium. The cells were then incubated for 48 h at 37 °C with 5% CO_2_. Stock solutions of the compounds were prepared individually using dimethyl sulfoxide (DMSO). Following incubation, the cell culture medium was carefully aspirated, and the cells were washed twice with 1× PBS. Subsequently, 50 µL of fresh XTT labeling reagent (Cell Proliferation Kit II, Merck Sigma-Aldrich) was added to each well and incubated for 4 h at 37 °C with 5% CO_2_. Then, the absorbance at 570 nm was measured using a Varioskan LUX multimode microplate reader (Thermo Fisher Scientific, Waltham, MA, USA). The CC_50_ (50% cytotoxic concentration), representing the concentration of the compound causing a 50% reduction in cell viability or inhibition of cell proliferation was determined using GraphPad Prism v.9.2.0 (GraphPad Software, La Jolla, CA, USA). All experiments were conducted in triplicate.

### 2.11. Drug Susceptibility Assay Based on Replication Component SARS-CoV-2

The susceptibility of the SARS-CoV-2 isolate hCoV-19/New Zealand/NZ1_patient/2020 to remdesivir, ebselen, and their spray-dried powder formulations was assessed using Calu-3 cells as described [37]. Serial dilutions spanning empirically determined ranges of each compound were added in triplicate to 96-well plates containing Calu-3 cells (20,000 cells/well). The plates were then incubated at 37 °C with 5% CO_2_ for two hours. Afterward, the cells were infected with SARS-CoV-2 at a multiplicity of infection (MOI) of 0.005 IU/cell for one hour at 37 °C with 5% CO_2_. Following virus inoculation, the viral inoculum was removed, cells were washed twice, and the complete medium containing the corresponding dilution of the test agent was replenished. SARS-CoV-2 replication was assessed 72 h post-infection using cytopathic effect (CPE) analysis and a cell protection assay based on the Pierce™ BCA Protein Assay Kit (Thermo Fisher Scientific), as mentioned earlier [37]. The concentrations of the compounds required to inhibit SARS-CoV-2 replication by 50% (EC_50_) were determined by plotting the percent inhibition of virus replication against the log10 of the drug concentration and fitting the inhibition curves to the data using nonlinear regression analysis with GraphPad Prism v.9.3.1 software (GraphPad Software).

### 2.12. Statistical Analysis

One-way ANOVA was performed using Tukey’s test as a post hoc test (*p* < 0.05) and using the Instat Graph Pad Prism (version 5, USA). The data are presented as mean ± SD (standard deviation).

## 3. Results and Discussion

### 3.1. Yield, Particle Size, and Residual Solvent

Table 1 represents the yield, particle size, and residual solvent of the prepared dry powders. The yield was >32% for all the dry powders under the mentioned spray-drying conditions. The highest yield obtained was ~56% for the spray-dried remdesivir (SD_R), whereas the lowest yield was ~33% for the spray-dried ebselen (SD_E). The yield of the dry powder formulation (SD_RE) comprising remdesivir and ebselen was about 34%. The dry powder containing a combination of remdesivir, disulfiram, and L-leucine (SD_REL) showed a higher yield (~40%) than SD_RE. The L-leucine-containing dry powder generally showed a higher yield, as the incorporation of leucine in the powder formulation reduces the powder cohesiveness, which ensures better powder deposition in the spray-dryer collector [38]. The yield of dry powder obtained using the spray-drying technique is generally low and lies between 20 and 50% [39]. The yield achieved in this study is comparable to the published report.

The deposition pattern of dry powder in the respiratory tract/lung is highly dependent on the particle size [34,40]. A bigger particle size is responsible for higher mass median aerodynamic diameter (MMAD) values. The particles with MMAD values between 1 and 5 μm are suitable for deep lung delivery, whereas the particles with more than 5 μm MMAD are mostly deposited in the extrathoracic part, and the particles with less than 1 μm are mostly exhaled [41]. The average particle sizes of the SD_R and SD_E were 1.7 μm and 2.3 μm, respectively. The SD_RE particle size was 1.6 µm, while the SD_REL particle size was 1.3 µm. SD_REL showed smaller particles than SD_RE. During the rapid spray-drying, the hydrophobic leucine migrates on the powder surface, which affects the particle size [38]. The particle sizes were statistically significant (*p* < 0.05) to each other except for SD_R and SD_RE. The median diameters of the size distribution (D_50_) values were obtained to be 1.6 μm, 2.1 μm, 1.5 μm, and 1.2 μm for SD_R, SD_E, SD_RE, and SD_REL, respectively. D_50_ means that 50% of the particle size is below the value, and the remaining 50% is above that value.

The amount of residual solvent present in all the dry powders was ~1% (*w*/*w*). A low water content (<5% *w*/*w*) is preferable for better aerosol performance [39,42]. The water content >5% (*w*/*w*) in the dry powder may impair the aerosol performance and stability [39,42]. The hydrophobic nature of the drugs and the use of organic solvent (methanol) could explain the low amount of solvent. During the HPLC, no additional peaks were observed, confirming the feasibility of the preparation procedure.

### 3.2. Powder Morphology

**Figure 1:**
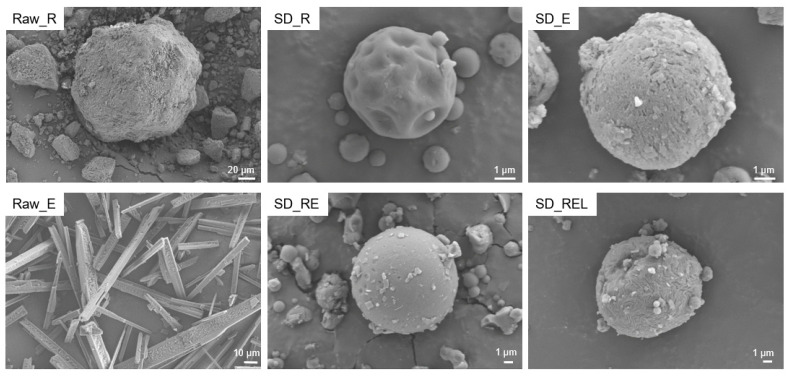
Representative scanning electron microscopy images of remdesivir raw material (Raw_R), ebselen raw material (Raw_E), spray-dried remdesivir (SD_R), spray-dried ebselen (SD_E), spray-dried remdesivir/ebselen combination (SD_RE), and spray-dried remdesivir/ebselen/L-leucine combination (SD_REL).

Figure 1 represents the morphology of the raw materials and dry powder formulations. The aerosol performance of dry powder is dependent on the powder morphology, as the shape of the powder can impact the drag and terminal velocities of the dry powder during aerosolization [43]. Remdesivir raw material (Raw_R) was flaky, whereas ebselen raw material (Raw_E) was acicular in shape. All the prepared dry powders were spherical in shape. SD_R showed dimples on the surface, but SD_E showed a dimple-free surface. SD_RE had a smooth surface, whereas leucine-containing SD_REL showed roughness on the surface.

### 3.3. Drug–Drug/Drug–Excipient Interaction

**Figure 2:**
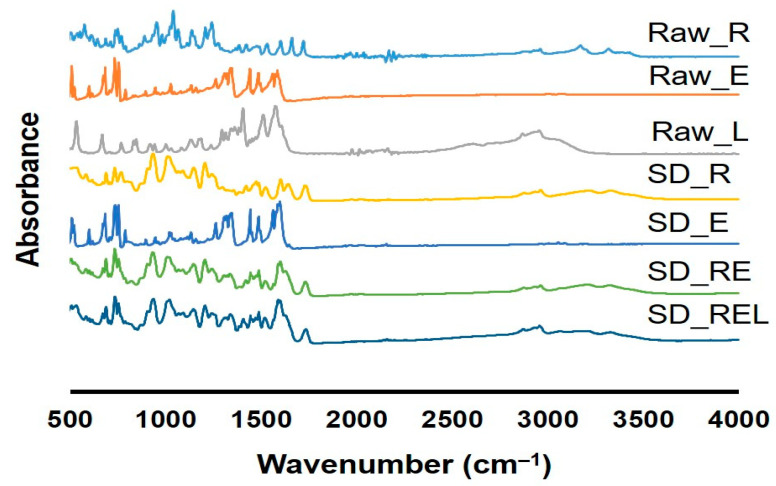
ATR-FTIR of supplied remdesivir (Raw_R), ebselen (Raw_E), L-leucine (Raw_L), spray-dried remdesivir (SD_R), spray-dried ebselen (SD_E), spray-dried remdesivir/ebselen combination (SD_RE), and spray-dried remdesivir/ebselen/L-leucine combination (SD_REL).

There were no drug–drug/drug–excipient interactions observed, as all the prepared dry powders showed similar peaks in the ATR-FTIR study corresponding to the raw materials (Figure 2). Similar peak positions of remdesivir and ebselen in raw materials (Raw_R and Raw_E) were found in their respective spray-dried dry powders (SD_R, SD_RE, and SD_REL). For example, the peak positions for Raw_R observed at 1661 cm^–1^ (representing the imine group), 1040 (representing the amine group), and 1603 (representing the aromatic ring) were also found in its spray-dried powder. Similarly, the peak positions for Raw_E observed at 896 cm^–1^ (representing selenium), 1263 cm^–1^ (representing tertiary amine), 1561 cm^–1^ (representing aromatic ring), and 1585 cm^–1^ (representing carbonyl group) were also observed in its spray-dried powder.

### 3.4. Powder Crystallinity

**Figure 3:**
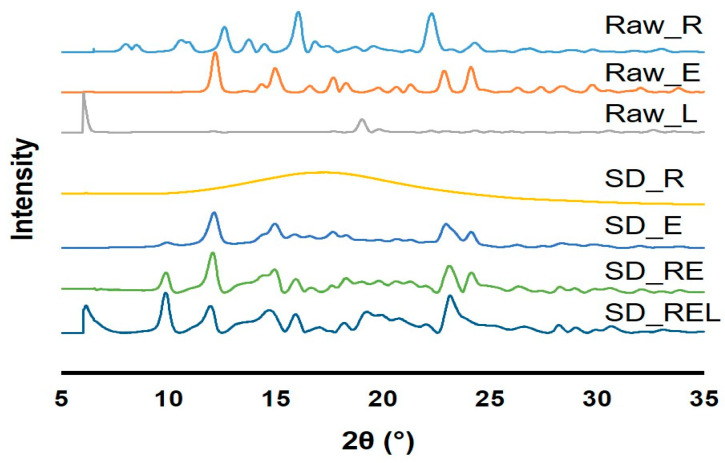
X-ray diffractograms of supplied remdesivir (Raw_R), ebselen (Raw_E), L-leucine (Raw_L), spray-dried remdesivir (SD_R), spray-dried ebselen (SD_E), spray-dried remdesivir/ebselen combination (SD_RE), and spray-dried remdesivir/ebselen/L-leucine combination (SD_REL).

The X-ray diffractogram (XRD) of raw materials and prepared dry powders are shown in Figure 3. The XRD peaks confirm that all of the raw materials and manufactured dry powders, with the exception of SD_R, were crystalline in form. The crystalline nature of remdesivir, ebselen, and L-leucine has already been reported [17,21,44]. The study also reported the amorphous character of remdesivir dry powder; however, the manufacturing method was different from ours [17]. The researchers used a thin film freezing technique to prepare dry powder of remdesivir, whereas a spray-drying technique was used in this study. Crystalline dry powders are more stable than amorphous dry powders [45]. Because the amorphous dry powder dissolves fast in the lung epithelium, inhalation may cause a cough, which is undesirable in infectious disorders such as COVID-19 [45]. In that regard, manufactured crystalline dry powders are advantageous.

### 3.5. In Vitro Aerosolization Behavior

**Figure 4:**
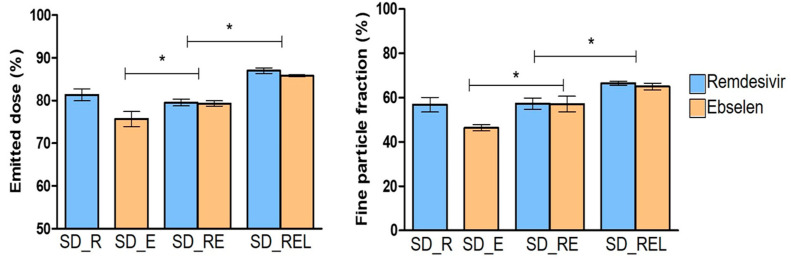
Emitted dose and fine particle fraction of spray-dried remdesivir (SD_R), spray-dried ebselen (SD_E), spray-dried remdesivir/ebselen combination (SD_RE), and spray-dried remdesivir/ebselen/L-leucine combination (SD_REL). (* indicating *p* < 0.05).

The in vitro aerosolization of the prepared dry powder formulation is shown in Figure 4. SD_E showed the lowest ED and FPF of 76% and 43%, respectively, while SD_R and SD_RE showed a similar ED and FPF of ~80% and ~58%. The reason could be the similar particle sizes of SD_R (1.7 μm) and SD_RE (1.6 μm). The ED and FPF of SD_RE were significantly higher than SD_E. The L-leucine-containing dry powder of remdesivir and ebselen (SD_REL) showed the highest ED and FPF of >85% and >65%. The ED and FPF increased significantly in the SD_REL compared with the SD_RE. The reason for these high ED and FPF values could be the incorporation of L-leucine, as it can enhance both the ED and FPF by producing a less cohesive powder with roughness on the particle surface [46,47]. L-leucine generally reduces the surface energy of the dry powders, which lowers the interparticle cohesion, resulting in improved aerosolization [48].

### 3.6. In Vitro Cellular Toxicity and Anti-SARS-CoV-2 Activity of the Dry Powder Formulation

**Figure 5:**
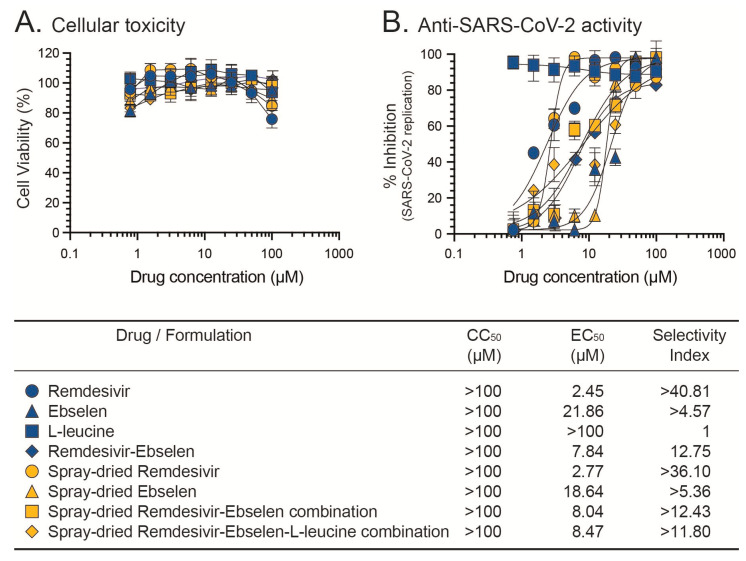
In vitro (**A**) cytotoxicity and (**B**) anti-SARS-CoV-2 activity of raw materials—remdesivir, ebselen, L-leucine, combination of raw remdesivir plus ebselen, spray-dried remdesivir, spray-dried ebselen, spray-dried remdesivir/ebselen combination, and spray-dried remdesivir/ebselen/L-leucine combination. Cellular toxicity (CC_50_ values) and susceptibility of SARS-CoV-2 (EC_50_ values) to the different individual or combinations of antiviral agents was evaluated in Calu-3 cells. SARS-CoV-2 replication was quantified 72 h post-infection by CPE and using a cell protection assay based on the Pierce™ BCA Protein Assay Kit (Thermo Fisher Scientific). Selectivity index (CC_50_/EC_50_) values are indicated. Results from raw or spray-dried antiviral drugs are indicated in blue or yellow, respectively.

The cytotoxic effect of the active drugs (raw materials) (i.e., Raw_R and Raw_E), excipient (Raw_L), and single as well as combined spray-dried formulations (SD_R, SD_E, SD_RE, and SD_REL) were assessed for cell viability and proliferation. No cytotoxicity was observed when the Calu-3 cells were exposed to either the active drugs, excipient, or the spray-dried formulations (CC_50_ > 100 μM, Figure 5). It is worth noting that both Raw_R and Raw_L exhibited similar CC_50_ values (>100 μM) in the Calu-3 cells, as reported in previous studies [21,49], although ebselen has shown limited cellular toxicity in Vero E6 cells (CC_50_ ~30 μM and 37.5 μM) [50,51]. The in vitro anti-SARS-CoV-2 activity of the single and/or combined spray-dried formulations was also determined. The antiviral activity of SD_R was similar to that of Raw_R (EC_50_ values 2.77 vs. 2.45 µM), and SD_E showed comparable antiviral activity to Raw_E, i.e., EC_50_ values of 21.86 vs. 18.64 µM (Figure 5). The spray-dried powders, both the L-leucine-free and L-leucine-containing powders (SD_RE and SD_REL), displayed similar antiviral effects against SARS-CoV-2, with EC_50_ values of 8.04 µM vs. 8.47 µM, respectively. Interestingly, previous studies described comparable EC_50_ values for raw remdesivir (EC_50_ values ranging from 0.83 to 1.65 µM) [37,52] or raw ebselen (EC_50_ values ranging from 4.67 to 34 µM) depending on the cell line used [51,53,54]. More importantly, the spray-dried combination of remdesivir and ebselen showed a similar in vitro anti-SARS-CoV-2 activity to the combination of raw remdesivir plus ebselen (EC_50_ values 8.04 vs. 7.84 µM, respectively). These findings suggest that the prepared spray-dried formulations retained a similar potency as the raw materials in inhibiting the replication process of SARS-CoV-2 in human airway epithelial Calu-3 cells.

## 4. Conclusions

In this study, we developed an inhalable dry powder containing a combination of remdesivir and ebselen utilizing a spray-drying technique. The microparticles exhibited a spherical shape with a crystalline nature, and L-leucine enhanced the emitted dose and fine particle fraction of the dry powder significantly. Importantly, the anti-SARS-CoV-2 properties of the prepared dry powders remained similar to the active drugs, and a high cell viability in the Calu-3 cells was observed, indicating their suitability for respiratory tract delivery to inhibit SARS-CoV-2. Further stability studies of the prepared dry powders in different storage conditions and in vivo studies in the appropriate animal models (e.g., humanized mice, ferrets, and/or African green monkeys) will ensure the suitability of the prepared dry powder formulations. Currently, numerous studies are focusing on inhalable formulations of single anti-SARS-CoV-2 agents. However, the approach described in this study, based on a combinational dry powder formulation with two drugs of different mechanisms of action, could be used as an initial road map to not only treat but also prevent SARS-CoV-2 infections.

## Figures and Tables

**Table 1:** The yield, particle size, and residual solvent amount of spray-dried remdesivir (SD_R), spray-dried ebselen (SD_E), spray-dried remdesivir/ebselen combination (SD_RE), and spray-dried remdesivir/ebselen/L-leucine combination (SD_REL).

Formulation	Remdesivir/Ebselen/L-Leucine (Molar Ratio)	Yield (%)	Particle Size * (μm)	D_50 (_μm)	Residual Solvent (%)
SD_R	1:0:0	55.6	1.7 ± 0.6	1.6	1.2 ± 0.0
SD_E	0:1:0	32.8	2.3 ± 0.8	2.1	0.2 ± 0.1
SD_RE	1:1:0	33.9	1.6 ± 0.7	1.5	0.8 ± 0.0
SD_REL	1:1:1	39.8	1.3 ± 0.5	1.2	0.9 ± 0.1

* Particle sizes are statistically significant (*p* < 0.05) to each other except for SD_R and SD_RE.

## Data Availability

Raw data can be requested by contacting the corresponding authors.
